# Epithelial-to-Mesenchymal Transition in Diabetic Nephropathy: Fact or Fiction?

**DOI:** 10.3390/cells4040631

**Published:** 2015-10-09

**Authors:** Ivonne Loeffler, Gunter Wolf

**Affiliations:** Department of Internal Medicine III, University Hospital, University of Jena, Erlanger Allee 101, D-07747 Jena, Germany

**Keywords:** epithelial-to-mesenchymal transition (EMT), endothelial-to-mesenchymal transition (EndoMT), diabetic nephropathy, renal fibrosis

## Abstract

The pathophysiology of diabetic nephropathy (DN), one of the most serious complications in diabetic patients and the leading cause of end-stage renal disease worldwide, is complex and not fully elucidated. A typical hallmark of DN is the excessive deposition of extracellular matrix (ECM) proteins in the glomerulus and in the renal tubulointerstitium, eventually leading to glomerulosclerosis and interstitial fibrosis. Although it is obvious that myofibroblasts play a major role in the synthesis and secretion of ECM, the origin of myofibroblasts in DN remains the subject of controversial debates. A number of studies have focused on epithelial-to-mesenchymal transition (EMT) as one source of matrix-generating fibroblasts in the diseased kidney. EMT is characterized by the acquisition of mesenchymal properties by epithelial cells, preferentially proximal tubular cells and podocytes. In this review we comprehensively review the literature and discuss arguments both for and against a function of EMT in renal fibrosis in DN. While the precise extent of the contribution to nephrotic fibrosis is certainly arduous to quantify, the picture that emerges from this extensive body of literature suggests EMT as a major source of myofibroblasts in DN.

## 1. Introduction

Diabetic nephropathy (DN) is one of the most serious complications in diabetic patients and the primary cause of end-stage renal failure. It develops in up to 30%–40% of patients with types 1 or 2 diabetes mellitus and is characterized by the accumulation of extracellular matrix (ECM) proteins, including predominantly various collagens, laminin and fibronectin in the mesangium and renal tubulointerstitium as well as thickening of basement membranes [1,2,3]. This increased deposition of ECM subsequently leads to renal fibrosis, which can culminate in tubulointerstitial fibrosis, glomerulosclerosis, infiltration of inflammatory mediators, and the *de novo* activation of alpha-smooth muscle actin (α-SMA)-positive myofibroblasts [1,3,4]. It has been shown that the number of myofibroblasts is inversely correlated with renal function in human DN [5,6]. It is widely accepted that these activated myofibroblasts are the principal effector cells that are responsible for the excess deposition of interstitial ECM under pathological conditions, but their origin is still a topic of hot debate [3,7]. The relevant mechanisms involved in the activation process of the matrix-producing myofibroblasts in the fibrotic kidney have been extensively investigated. The first causal association of fibrosis with EMT stems from the observation that epithelial cells may express fibroblast markers and undergo phenotypic changes reminiscent of fibroblasts in disease states [7,8]. The aberrant expression of fibroblast-specific protein in renal tubular epithelial cells led Strutz *et al.* to postulate that some myofibroblasts might be derived from transformed epithelial cells [8,9]. The interest in renal epithelial-to-mesenchymal transition (EMT) increased further with the confirmation of this hypothesis, when Iwano *et al.* showed that up to 36% of all myofibroblasts can arise via local EMT from tubular epithelial cells during kidney fibrosis [10]. *In vitro* studies have shown that a multitude of agents can trigger EMT, prominently amongst them the profibrotic protein transforming growth factor-β1 (TGF-β1) [11]. The relevance of EMT to pathologic renal fibrosis was further corroborated by Zeisberg *et al.,* who demonstrated that bone morphogenic protein 7 (BMP-7) counteracted TGF-β1-induced EMT and could reverse renal fibrosis both *in vitro* and *in vivo* [12]. Although EMT in renal fibrosis was originally postulated on the basis of purely correlative evidence, growing evidence has implicated this process as a major pathway leading to the generation of interstitial myofibroblasts in diseased kidneys [7]. The study of renal EMT further proliferated with nearly 600 articles published on this subject at present. During the last several years, substantial progress has been made in providing evidence for the existence and significance of EMT in DN, and several reviews, personal perspectives, and debates have been published in this field [2,4,5,13,14,15,16,17,18,19,20,21,22,23,24,25,26,27,28,29]. Newer findings indicating that activated myofibroblasts originate from multiple lineages has subsequently cast doubts on the contribution of EMT to renal fibrosis *in vivo* and more importantly, has led many to speculate on the exact cell type involved [4]. Moreover, sparked by the elegant study of Humphreys *et al.* in 2010, which found no evidence for EMT in the gold-standard murine model of renal fibrosis [30], other investigators subsequently raised doubts regarding the occurrence and relevance of EMT *in vivo* [20]. At present, the importance of EMT for renal fibrosis is a subject of heated debate. This review tries to summarize and dissect the evidence for and against EMT and discuss whether EMT is a direct contributor to the development of renal fibrosis in DN. In addition, we will also discuss and review literature data concerning endothelial-to-mesenchymal transition (EndoMT), the analogous process to EMT in endothelial cells.

## 2. The Origin of Myofibroblasts in the Fibrotic Kidney

**Figure 1:**
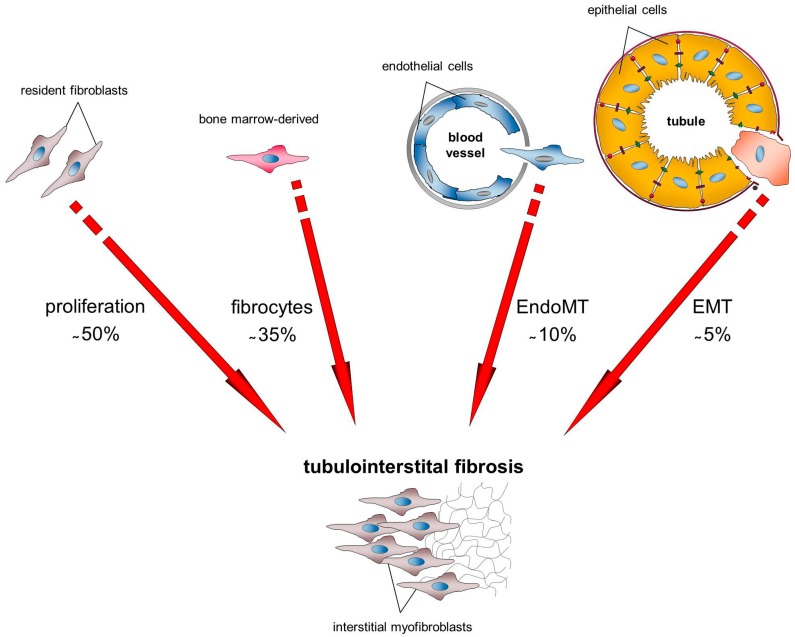
Current concept of origin of matrix-producing myofibroblasts that directly mediate the tubulointerstitial fibrosis. Schematic illustration shows the possible mechanisms via myofibroblasts can originate during kidney fibrosis with their proposed proportions on the total number of interstitial myofibroblasts. Recent studies estimate that approximately 35% of fibroblasts arise from bone marrow, 10% and 5% via local EndoMT or EMT, respectively, and 50% of fibroblasts result from proliferation of resident fibroblasts [32]. EndoMT (endothelial-to-mesenchymal transition), EMT (epithelial-to-mesenchymal transition).

While it is widely accepted that the matrix-producing myofibroblasts in the renal interstitium are the major source of the increased ECM, their exact origin and activation process in the fibrotic kidney remains largely undefined and controversial [7,31]. A crucial advance in our understanding of renal fibrosis is that multiple cell types are responsible for the accumulation and remodeling of ECM [13]. For example, studies of models of kidney fibrosis have demonstrated that myofibroblasts can be derived from bone marrow, tubular epithelium, and vascular endothelium [16]. In the literature, controversial opinions about the origin of myofibroblasts are discussed: some studies favor the idea that vascular pericytes may serve as precursors to myofibroblasts in fibrosis, others have disputed the contribution of the EMT and EndoMT in the emergence of myofibroblasts and fibrosis, and a suggestion that bone marrow contributes to the total myofibroblast population has also been put forward [32]. However, determining the origins of renal myofibroblasts remains important because this may account for the well-known heterogeneous characteristics and behaviors of myofibroblasts [33]. The controversy about their sources and their contribution to fibrosis are clearly presented in the review from Mack *et al.* [33]. The differences in the findings are quite serious that, for example, Lin *et al.* [34] published that less than 0.1% of all myofibroblasts are bone marrow-derived cells, whereas LeBleu *et al.* [32] found that 35% are fibrocytes derived from bone marrow. The same dramatic discrepancies are found regarding the contribution of epithelial cells to kidney fibrosis via the EMT mechanism, spanning a range from 36% [10], approximately 5% [32], to almost no contribution [30]. Of particular note is that nearly all data were generated using the model of unilateral ureteral obstruction (UUO) because this model recapitulates various key features of fibrotic responses together with tubular damage and is therefore the most intensively used model to study fibrosis in the kidney [33]. However, current data strongly suggest that collagen-producing myofibroblasts in the kidney can be derived from various cellular sources, including epithelial cells, endothelial cells, and bone marrow endosteal cells [3,33]. Figure 1 shows the current view of the experts about the origin of the myofibroblasts in the fibrotic kidney based on the experiments from LeBleu and coworkers. They used multiple genetically engineered mice to track, fate map, and ablate cells to determine the source and function of myofibroblasts in kidney fibrosis 10 days after UUO and identified that the total pool of myofibroblasts is heterogeneous. The accumulation of myofibroblasts occurs predominantly from two different sources: 1) with 50% through the local proliferation of resident fibroblasts; and 2) with 35% the contribution of bone marrow-derived cells without any evidence of proliferation in the kidney [32]. In addition, their fate-mapping experiments showed that a significant number of tubular epithelial cells gain α-SMA expression in fibrosis and 5% of myofibroblasts are truly derived through a fully executed EMT. They also confirm that 10% of myofibroblasts are derived through a mechanism called EndoMT [32]. EndoMT is, similar to EMT, a process that is involved in fibrosis by ultimately increasing the number of glomerular and tubulointerstitial myofibroblasts [19,31]. During EndoMT, endothelial cell markers gradually diminish while the transitioning cells acquire mesenchymal markers [5]. Incidentally, the hypothesis that pericytes play a critical role in the pathogenesis of tissue fibrosis has also become a controversial issue. In contrast to the data from Humphreys *et al.*, which demonstrate that more than 90% of myofibroblasts in UUO result from activated pericytes, LeBleu *et al.* found that pericytes do not contribute to kidney fibrosis in the same model [30,32,33]. A further origin of matrix-producing myofibroblasts in the fibrotic kidney can be the glomerular epithelial cells, the podocytes. Recent experimental evidence has supported the hypothesis that podocytes may undergo EMT after injury, leading to podocyte dysfunction that ultimately leads to defective glomerular filtration [35]. Taken together, it is increasingly clear that fibroblasts derive from multiple parental lineages in kidney fibrosis, but it is less investigated whether the current described proportion of the different cell types on the contribution to fibrosis, especially in DN, are equal or whether and to what proportion EMT is a source of interstitial fibroblasts in DN. There are several pieces of evidence suggesting that EMT occurs in the development of fibrosis in DN. For example, in patients with type 2 DN it has been shown that the location of α-SMA positive cells was always in the interstitial space, either around the tubular basement membrane (TBM) or in between its layers [24].

Beyond podocytes and glomerular endothelial cells, mesangial cells have been put forward as an additional precursor cell type of myofibroblasts on the basis of experimental and clinical studies of DN. The involved process has been termed myofibroblast transdifferentiation (MFT) and alters the normal phenotype of glomerular mesangial cells to produce myofibroblasts that secrete excess ECM [13]. Moreover, MFT is also believed to be the mechanism that bone marrow-derived progenitors undergo to become effector cells/myofibroblasts in renal fibrosis [13].

## 3. Transition of Renal Cells into Activated Myofibroblasts: The Mechanism

**Figure 2:**
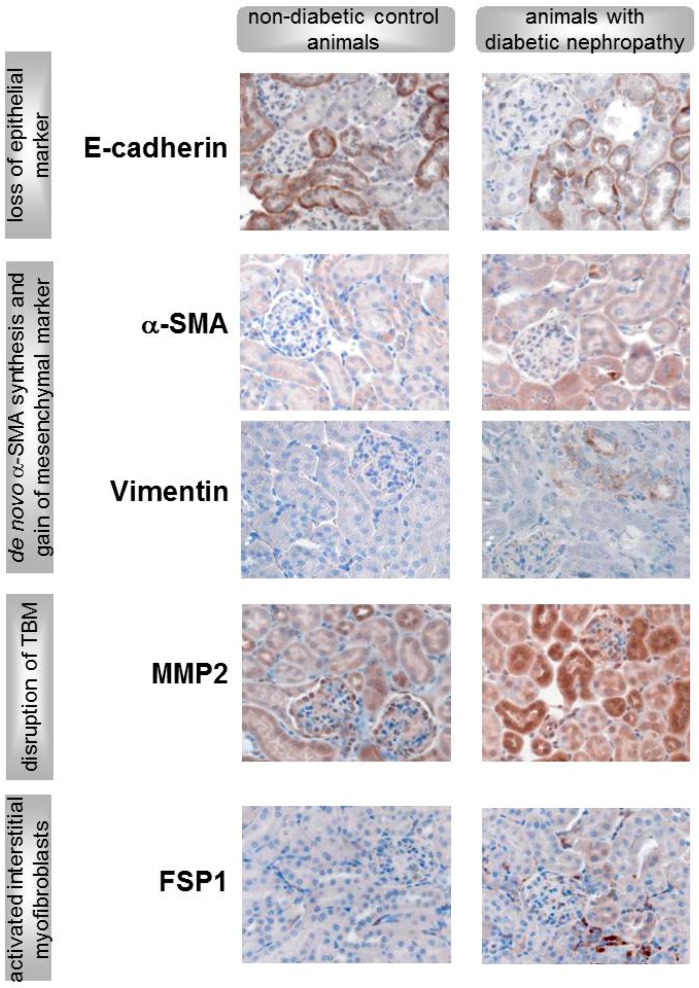
Evidence for tubular EMT in streptozotocin (STZ)-induced diabetic mice (unpublished data from the authors’ laboratory). Shown are representative immunhistochemical stainings reflecting the four key events of EMT leading to activated interstitial myofibroblasts: (1) reduction of epithelial marker (e.g. E-cadherin), (2) *de novo* α-SMA synthesis and increased expression of mesenchymal marker (e.g. vimentin), (3) disruption of tubular basement membrane (TBM) by matrix metalloproteinases (e.g. MMP2), and (4) invasion of activated myofibroblasts into the interstitium (FSP1 positive cells). Magnification 400x.

**Figure 3:**
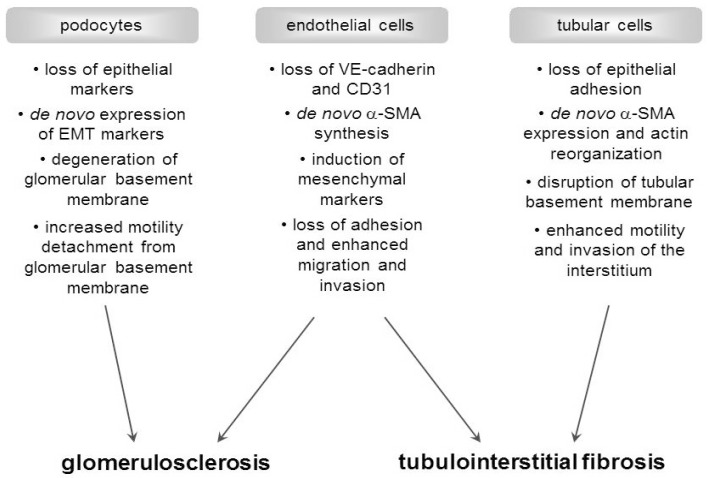
Hypothesized progression of fibrosis by EMT and EndoMT in the interstitium or in the glomerulus. The illustration shows four key events essential for the completion of EMT and EndoMT, which lead to glomerulosclerosis and tubulointerstitial fibrosis. For more details see the text [5,15,16,17,18,33,40].

Although it is controversially discussed in the literature whether or not EMT/EndoMT plays an important role in the development of fibrosis *in vivo*, there are no doubts that the transition of renal cells into matrix-producing fibroblasts does exist and is inducible *in vitro*. Several factors and pathways are known to drive these mechanisms, but of the many factors that regulate EMT or EndoMT in different ways, TGF-β1 is the most potent inducer capable of initiating and completing the entire processes [1,5,7,14,16,36,37,38]. EMT describes a complex set of phenotypical changes induced in epithelial cells that lose their defined cell-cell-basement membrane contacts and their structural polarity to become spindle-shaped and morphologically similar to mesenchymal/myofibroblast cells [39]. Three subtypes of EMT have been classified depending on the physiological tissue context: type I EMT occurs in embryogenesis and organ development; type II EMT is induced in the context of inflammation and fibrosis, and type III EMT is associated with cancer progression [38,39]. In all tissue contexts, key events in EMT are the dissolution of the epithelial cell-cell contacts, loss of apical-basal polarity, reorganization of the cytoskeletal architecture and changes in cell shape, downregulation of an epithelial gene expression signature and activation of genes that help to define the mesenchymal phenotype, and increased cell protrusions and motility [38]. However, type II EMT is commonly defined as the ability of adult epithelial cells to undergo dedifferentiation [4]. The transition of epithelial cells into mesenchymal cells follows a common and conserved program with hallmarks (Figure 2 and Figure 3) [38]. EMT is an orchestrated, highly regulated process defined by four key events: (1) loss of epithelial cell adhesion (e.g. modification of the E-cadherin adhesion complex), (2) *de novo* α-SMA expression and actin reorganization, (3) disruption of TBM by matrix metalloproteinases (MMPs), and (4) enhanced cell migration and invasion of the surrounding interstitium [7,40]. This migratory capacity leads to increased deposition of the ECM and makes EMT pivotal in the pathology of tubulointerstitial fibrosis [4]. The early stages of EMT are the disassembly of epithelial cell-cell contacts (tight junctions, adherens junctions, desmosomes, and gap junctions) and the loss of cell polarity [14,38]. The expression of epithelial genes is suppressed concomitantly with the activation of mesenchymal gene expression [38]. Next, the epithelial actin architecture reorganizes, the epithelial cells lose their cobblestone morphology and become elongated and spindle shaped, which is accompanied by the *de novo* expression of α-SMA [11,38]. As the epithelial cells are released from the basement membrane, they can invade the interstitium through gaps in the damaged basement membrane that is disrupted by matrix metalloproteinases, such as MMP2 and MMP9 [3]. It is believed that an intact TBM may be necessary to maintain the epithelial phenotype and its disruption is sufficient to trigger EMT *in vitro* [11]. As mentioned above, every event in the process of EMT is highly regulated and multiple intracellular signaling pathways have been implicated in mediating EMT [7]. Many studies have demonstrated that EMT progression is regulated by several signaling pathways or molecules that can cooperate to induce full EMT responses. For example, Smad, p38 MAPK, TLRs, Wnt, mTOR, Notch, small GTPase and Hedgehog pathways can participate in EMT [1,27,28,38,41]. Moreover, recent reports have established a role for miRNAs in the process of EMT, making the complexity of the regulation of EMT clear [42,43]. However, tubular EMT, by definition, is a process in which tubular cells lose their epithelial phenotype and acquire new characteristic features of mesenchyme [7]. The same is believed to occur with glomerular epithelial cells, the podocytes (Figure 3) [44]. Podocytes are highly specialized epithelial cells that cover the outer aspect of the glomerular basement membrane (GBM), playing a crucial role in the regulation of glomerular function [45]. Phenotypic changes of podocytes associated with an increase in podocyte motility and expression of new markers is documented in human diabetic kidney disease and many different animal models and appears to be a common theme associated with proteinuria [15]. Therefore, more recently, reports have recommended EMT as a new paradigm and potential explanation for podocyte depletion in DN [35,44,46]. Several research teams have suggested that endothelial cells may also acquire functional and structural characteristics of mesenchymal cells via EndoMT in different mouse models of chronic kidney disease including animal models of DN [17,19,31,47,48]. Similar to EMT, during EndoMT, endothelial cells lose their adhesion and apical-basal polarity to form highly invasive, migratory, spindle-shaped, elongated mesenchymal cells [49]. EndoMT is also a complex biological process in which tubulointerstitial vessels as well as glomerular endothelial cells lose their specific endothelial cell markers, such as vascular endothelial cadherin (VE cadherin) and CD31, and acquire a mesenchymal or myofibroblastic phenotype initiating expression of mesenchymal cell products such as α-SMA, vimentin, and collagen type I [50]. Besides the acquisition of an activated pro-fibrogenic phenotype, these cells also become motile and are capable of migrating into surrounding tissues (Figure 3) [50]. Similar to EMT, EndoMT can be induced by members of the TGF-β family of signalling proteins, hyperglycemia, advanced glycation end-products (AGEs), and angiotensin II [5,16]. Taken together, in the presence of fibrosis-promoting stimuli, cells undergo a number of morphological and phenotypic changes in which they lose cell type-specific characteristics in exchange for markers more commonly associated with a mesenchymal phenotype [14].

## 4. *In Vitro* Evidence for EMT and EndoMT of Renal Cells in the Context of Diabetic Conditions

A variety of cell-culture models have been used to examine the processes by which renal cells can undergo cellular transition. Tubular and glomerular EMT as well as EndoMT are elicited *in vitro* under various conditions, including stimulation with TGF-β1, high glucose, or AGEs. Zeisberg *et al.* recently suggested *in vitro* criteria that are useful parameters for recognizing EMT in cell culture: increased expression of cytoskeletal proteins, cadherin switch, new nuclear expression of several transcription factors, absence of epithelial markers, spindle-shape morphology and loss of polarity, increased migratory capacity, and resistance to apoptotic stimuli [51]. As listed in **Table 1**, data that point to EMT are variably based on the investigation of the different recommended criteria and respective key events. Investigators in most studies addressed the first two described key events of the EMT process by analysing, in different tubular epithelial cell lines or podocytes, the reduction of epithelial markers (E/P-cadherin and/or ZO-1) and the induction of mesenchymal proteins (α-SMA and/or vimentin) after 48–72 h of stimulation with TGF-β1, high glucose, or other triggers of diabetes [12,25,27,28,29,43,46,52,53,54,55,56,57]. Other studies focused more on changes in the expression and localization of transcription factors such as β-catenin, Snail, and Twist, and still others include the changes in the expression of ECM components such as collagens and/or fibronectin [27,29,35,43,58,59]. To show the other two key events in the EMT mechanism (disruption of the TBM, invasion of the activated myofibroblasts in the interstitium), some studies demonstrate the enhanced expression of MMPs or at least the consequence of an ongoing EMT process — that is, the activated myofibroblast expressing specific fibroblast markers such as FSP1 and HSP47 [35,58,60]. Particularly noteworthy is one study that demonstrates all four key events described for the EMT: human proximal tubular cells were stimulated with TGF-β1 and after three days the cells exhibited a decreased E-cadherin expression accompanied by *de novo* α-SMA expression and actin reorganization [40]. Furthermore, it has been shown that secreted MMP2 and MMP9 have disrupted a matrigel with 15 µm depth, which is somewhat analogous to native TBM matrix [40]. To provide proof that EMT was completely successful in this study, the myofibroblast morphology as well as the enhanced migration and invasion were checked [40]. The following studies are also worthy of mention, since they point to inhibition or reversal of the EMT. It could be shown that recombinant human BMP-7 reversed TGF-β1-induced tubular EMT and also inhibited the transition of podocytes [12,56]. In addition, hepatocyte growth factor (HGF) has been identified as a potent inhibitor of EMT — it exhibits a remarkable ability to block TGF-β1-induced EMT in human proximal tubular cells. Moreover, HGF interacts with PGE2, a mediator that has also been shown to reverse EMT [52,61]. Interestingly, in the presence of metformin, a drug clinically used as an insulin sensitizer, TGF-β1-treated canine kidney epithelial cells changed from scattered fibroblast-like shapes to a more packed cobblestone morphology, which is characteristic of a reversal of EMT [62]. In addition, metformin could also prevent the TGF-β1-induced accumulation of the EMT marker vimentin in the cytoplasm of these cells [62]. According to the latest research, more factors than were previously known play a role in the regulation of EMT. They have shown that in human proximal tubule cells, glucose-evoked increases in TGF-β1 reduce cell-cell adhesion via decreasing connexin-43 (CX43) production [63]. Connexins, a family of transmembrane proteins, form partnerships with multiple adhesion and structural proteins (e.g. ZO-1 and N-cadherin) and have been shown to be downregulated in podocytes, endothelial cells, and tubular cells *in vitro*, in diabetic mouse models, and human DN [64]. Interestingly, it has been found that visfatin, an adipokine that is elevated in patients with type 2 diabetes, inhibits the expression of CX43 in proximal tubular cells, illustrating the intricacy involved in understanding the *in vivo* situation of diabetic patients [65].

As shown, the *in vitro* evidence for EMT induced by different diabetic factors is compelling, but is examined in culture and out of biological context. Therefore, the question remains as to whether EMT in DN does exist *in vivo*.

## 5*. In Vivo* Evidence for EMT or EndoMT in DN

Evidence for type II EMT *in vivo* is more difficult to document because EMT by definition is a plastic process with various intermediate stages [51]. Nevertheless, many previous studies have suggested that EMT as well as EndoMT occur in diabetes and play a vital role in the progression of DN (**Table 2**). Similar to the *in vitro* studies, based on the use of diverse methods including immunohistochemistry and mRNA analysis different criteria for EMT have been highlighted. The most common animal model used to investigate the transition of renal cells in DN is streptozotocin (STZ)-induced diabetes in rodents, a reasonable model for type 1 diabetes mellitus with development of DN. However, studies investigating the occurrence of EMT in patients with DN were also performed. Back in 2001, Oldfield *et al.* demonstrated that in kidney sections of chronically diabetic rats, approximately 5% of tubules expressed α-SMA, suggesting that EMT occurs in the kidney in the context of experimental STZ-induced diabetes. Furthermore, they were able to show a clear α-SMA staining of an epithelial cell within an intact tubule in a renal biopsy of a human type 1 subject with nephropathy [25]. Another report described basal filaments organized in bunches in some tubular epithelial cells observed in kidney biopsies of type 2 diabetic patients. This is a strong argument in favor of EMT theory because it reflects the changes in the expression and organization of cytoskeleton components described as key events in the mechanism of EMT [24]. In addition, the reduction of specific epithelial cell markers (E-cadherin and ZO-1), in combination with *de novo* α-SMA synthesis and the elevation of mesenchymal markers, has been shown in a study from our laboratory [2] as well as several other studies of rodent and human DN biopsies [26,27,29]. Following a previous study conducted by Rastaldi *et al.* that demonstrated the association of EMT features with serum creatinine and the degree of interstitial damage [26], Zheng *et al.* investigated the correlation of urinary mRNA markers of EMT with the progression of DN in patients with type 2 DN. They showed that α-SMA, fibronectin, and MMP-9 were significantly increased in DN patients compared with healthy controls. These results support the findings of Rastaldi *et al.—*the degree of expression of these EMT markers increased as renal function declined in patients with various stages of DN. More precisely, the urinary mRNA levels of all tested target genes were significantly correlated with urinary albumin as well as blood urea nitrogen [66]. Comparable to the experimental design of the *in vitro* studies, many investigators, including ourselves, combine several EMT markers in their studies in order to cover as many of the EMT key events and *in vivo* criteria for EMT as possible, including the expression of MMPs and transcription factors (see also Figure 2) [2,27,35,66]. Podocyte injury is important in DN progression. The hypothesis that podocytes undergo EMT, leading to podocyte dysfunction that ultimately results in defective glomerular filtration, is supported by several studies in animals and observations in humans. Recent studies demonstrated that podocytes changed phenotypically in the early stages of DN in STZ-induced rodents with increased mesenchymal markers (desmin) and decreased epithelial markers (nephrin) [35,46]. In addition to animal studies, the extensive reduction of nephrin and ZO-1 expression has also been observed in glomeruli of both patients with type 1 and type 2 diabetes [35,44,67]. A very conclusive study, done in human DN and covering several *in vivo* criteria, has been performed by Li *et al.* They demonstrate, with immunhistochemical stainings, a podocyte-specific attenuation of nephrin and ZO-1 as well as an enhancement of the expression of desmin, MMP9, and FSP1 [35].

Yamaguchi *et al.* found FSP1-positive podocytes in urinary excretion and renal biopsy specimens of patients with type 2 diabetes and found that the rise in FSP1 expression in the podocytes of diabetic patients is associated with more severe clinical and pathological findings [44].The following studies provide strong evidence suggesting that cells with an endothelial origin contribute to the early development of renal interstitial fibrosis in STZ-induced DN. The study of Zeisberg *et al.* is based on genetic lineage-tracing of endothelial cells and double-labeling of tissue for endothelial and fibroblast markers (FSP1/CD31 and α-SMA/CD31) in a six month STZ mouse model. They revealed that approximately 40% of all FSP1-positive cells and 50% of the α-SMA-positive cells in STZ kidneys were also CD31 positive, suggesting that these fibroblasts are likely of endothelial origin [19]. Li *et al.* also suggest that EndoMT is a novel mechanism that contributes to the generation of both interstitial and glomerular myofibroblasts in the DN [17]. Using an endothelial lineage-traceable mouse line, the authors demonstrated that after one month 10% and after 6 months approximately 25% of α-SMA-positive renal interstitial fibroblasts in STZ-induced diabetic kidneys were of endothelial origin [17]. In a later study, Li *et al.* showed that a Smad3 inhibitor delays the early development of DN and AGE-induced EndoMT [47]. These studies have provided evidence that approximately 25% to 50% of fibroblasts detected in the mouse models of diabetic nephropathy coexpressed the endothelial marker with markers of fibroblasts/myofibroblasts, such as FSP1 and α-SMA.

**Table 1:** Overview of the experimental designs of the major studies that demonstrated evidence for EMT *in vitro.*

Model	EMT Inducer Used	EMT Marker Investigated	Key Events	Study
renal epithelial cells	TGF-β1 (3 ng/mL; 48h)	ZO-1, cytokeratines, syndecan-1, α-SMA, vimentin, FSP-1	1, 2, (4)	[60]
human tubular epithelial cells	TGF-β1 (4 ng/mL; 72h)	E-cadherin, α-SMA, MMPs, disruption of TBM, migration and invasion	1, 2, 3, 4	[40]
mouse tubular epithelial cells	TGF-β1 (3 ng/mL; 48h)	E-cadherin, ZO-1	1	[12]
renal epithelial cells	TGF-β1 (10 ng/mL; 72h)	E-cadherin, α-SMA	1, 2	[52]
renal epithelial cells	TGF-β1 (5 ng/mL; 48h)	E-cadherin, ZO-1, α-SMA, vimentin	1, 2	[53]
tubular epithelial cells	TGF-β1 (5 ng/mL; 72h)	E-cadherin, β-catenin, α-SMA, HSP47	1, 2, 4	[58]
human tubular epithelial cells	TGF-β1 (10 ng/mL; 72h)	E-cadherin, vimentin	1, (2)	[28]
rat tubular epithelial cells	TGF-β1, TGF-β2 (10 ng/mL; 72h)	E-cadherin, α-SMA, vimentin, collagens	1, 2	[43]
tubular epithelial cells	TGF-β1 (10 ng/mL; 3 and 6 days)	E-cadherin, α-SMA	1, 2	[54]
rat tubular epithelial cells	high glucose (20 mmol/l; 12–72h)	E-cadherin, CK-18, α-SMA, fibronectin	1, 2	[29]
human and rat tubular epithelial cells	high glucose (60 mmol/l; 72h)	E-cadherin, Snail, twist, α-SMA	1, 2	[27]
rat renal epithelial cells	AGE-BSA (6 days)	E-cadherin, α-SMA	1, 2	[25]
rat tubular epithelial cells	AGE-BSA (3 and 6 days)	E-cadherin, α-SMA	1, 2	[54]
rat tubular epithelial cells	albumin (2–6 days)	E-cadherin, α-SMA, FSP-1	1, 2, (4)	[57]
human tubular epithelial cells	TGF-β1, high glucose, albumin, angiotensin II, aldosterone	E-cadherin, α-SMA	1, 2	[55]
mouse podocytes	TGF-β1 (0.5–5 ng/mL; 6–72h)	P-cadherin, ZO-1, nephrin, desmin, collagen I, fibronectin, MMP9	1, 2, 3	[35]
mouse podocytes	high glucose (30 mmol/l)	desmin	2	[46]
mouse podocytes	high glucose (25 mmol/l)	synaptopodin, desmin	1, 2	[56]

The numbers in brackets mean that key events are not directly addressed but inferred from reported findings. EMT: epithelial-to-mesenchymal transition, TGF-β1: transforming growth factor β1, ZO-1: zonula occludens 1, α-SMA: α-smooth muscle actin, FSP-1: fibroblast specific protein 1, MMP: matrix metallo-proteinase, TBM: tubular basement membrane, AGE: advanced glycation end-products, HSP47: heat shock protein 47.

**Table 2:** Overview of the experimental design of the major studies that demonstrated evidence for tubular and glomerular EMT and EndoMT *in vivo.*

Model	EMT/EndoMT	EMT Marker Investigated	Key Events	Study
STZ-induced rats	tubular EMT	α-SMA	2	[25]
human DN	tubular EMT	ZO-1, α-SMA, vimentin, collagens	1, 2, (4)	[26]
STZ-induced rats	tubular EMT	α-SMA, collagen IV	2	[54]
STZ-induced mice	tubular EMT	α-SMA, collagen IV, fibronectin	2	[43]
human DN	tubular EMT	bunches in tubular epithelial cells, interstitial myofibroblasts	2, (4)	[24]
STZ-induced mice	tubular EMT	E-cadherin, ZO-1, α-SMA, vimentin, MMP2, FSP1, collagens, fibronectin, integrins, Twist, ZEB1	1, 2, 3, (4)	[2]
STZ-induced rats	tubular EMT	E-cadherin, α-SMA, fibronectin	1, 2	[29]
human DN	tubular EMT	α-SMA, fibronectin, MMP9	2, 3	[66]
STZ-induced rats	tubular EMT	E-cadherin, Snail, twist, α-SMA	1, 2	[27]
human DN	glomerular EMT	Nephrin	1	[67]
STZ-induced mice	glomerular EMT	nephrin, desmin, MMP9	1, 2, 3	[35]
human DN	glomerular EMT	ZO-1, nephrin, desmin, MMP9, FSP1	1, 2, 3, (4)	[35]
human DN	glomerular EMT	ZO-1, Snail, FSP1	1, (4)	[44]
STZ-induced mice	glomerular EMT	nephrin, synaptopodin	1	[68]
STZ-induced rats	glomerular EMT	nephrin, desmin	1, 2	[46]
STZ-induced mice	EndoMT	endothelial lineage tracing	1, 2, 3, 4	[19]
STZ-induced mice	EndoMT	endothelial lineage tracing	1, 2, 3, 4	[17]
STZ-induced mice	EndoMT	endothelial lineage tracing	1, 2, 3, 4	[47]

The numbers in brackets mean that key events are not directly addressed but inferred from reported findings. EMT: epithelial-to-mesenchymal transition, EndoMT: endothelial-to-mesenchymal transition, DN: diabetic nephropathy, ZO-1: zonula occludens 1, α-SMA: α-smooth muscle actin, FSP-1: fibroblast specific protein 1, MMP: matrix metallo-proteinase.

## 6. Evidence against the EMT Theory as One Source for Matrix-Producing Myofibroblasts

The only available evidence in the literature speaking against EMT uses general models of kidney fibrosis (for example, UUO and ischemia-reperfusion) and does not address DN specifically. Nevertheless, in the interests of a comprehensive review, the findings of the most important of these studies will be highlighted here. In 2010, the contribution of EMT to renal interstitial fibrosis *in vivo* was challenged by experimental data generated using cell lineage tracking techniques [30]. By tracking renal tubular epithelial cells *in vivo*, Humphreys *et al.* found no evidence to suggest that epithelial cells migrate outside of the tubular basement membrane and differentiate into interstitial myofibroblasts in two types of injury (UUO and ischemia-reperfusion) [30]. Furthermore, the authors did not detect any tubular cells that expressed α-SMA, implying that EMT did not occur [30]. This study provides strong evidence against the significance of the involvement of EMT in interstitial fibrosis in these two models [69]. In the same year, another group showed in a tetracycline-controlled transgenic mouse model that overexpression of TGF-β1 in renal tubules induces widespread peritubular fibrosis and that the fibrosis seen in between intact tubules and in areas of tubular decomposition resulted from myofibroblasts that were mainly derived from local fibroblasts and not via the transition of tubular epithelial cells [70]. Five years prior to the publication of these studies, in 2005, doubts regarding the EMT theory were reported in a study investigating the origin of interstitial fibroblasts in an accelerated model of angiotensin II-induced renal fibrosis. No evidence was found for an epithelial origin of interstitial myofibroblasts [71]. A further contradiction to the hypothesis that a multistep EMT program is necessary for fibrogenesis to occur has been demonstrated by the observation that TGF-β1/CTGF-mediated changes in E-cadherin expression in renal cells can occur independently of matrix protein synthesis, suggesting that the entire ‘classic EMT’ program, may not be critical for the development and progression of renal fibrosis [42]. Even current supporters of EMT have published evidence against EMT: although Mandache *et al.* have clearly seen, using electron microscopy, the presence of basal bundles of filaments in some tubular epithelial cells of kidney biopsies from patients with type 2 DN, they did not find α-SMA-positive tubular epithelial cells, which has been described as the most important key event in EMT [24]. Another study that argued against the hypothesis of EMT in human renal disease focused on the expression of myofilaments in tubular epithelial cells, endothelial cells, and interstitial cells [72]. Using light and electron microscopy, they found a widespread presence of actin in these cells, but no evidence of intermediate forms or migration of cells and therefore they believe that epithelium and interstitium could be separate targets for actin upregulation [72].

The ability to permanently tag cells *in vivo* has provided additional insights and the recent observations using lineage tracing technology strikingly point to the possibility that EMT is an artificial event that would never take place *in vivo*.

## 7. Discussion

The pivotal role of EMT in renal fibrosis was first put forward in an article published in JASN in 2011 [23]. At that time more than 300 original publications with evidence favoring EMT playing a role in the setting of fibrogenesis stood against only 5 that failed to detect EMT *in vivo*. Currently (August 2015), a pubmed search using the keywords ‘epithelial-to-mesenchymal transition’ and ‘kidney fibrosis’ yields almost 600 hits, and using the keywords ‘epithelial-to-mesenchymal transition’ and ‘DN’ at least 100, from which 80 are from the last 5 years. This large volume of publications, including original articles and reviews, illustrates that there is still interest in this issue. Although the existence of EMT in renal fibrosis is far from being unambiguously proven, many investigators tacitly take EMT as a given and proven phenomenon. Understandably, many investigators have selected specific aspects of the full EMT process depending on their particular focus of research. Critics of the role of EMT, such as Jeremy Duffield, have argued that tracing studies, the state-of-the-art method for the study of EMT *in vivo*, have failed to demonstrate epithelial migration and differentiation into myofibroblasts [23]. Although it is widely accepted that the lineage-tracing experiments allow the detection of a fibroblast’s origin within the kidney, the central challenge continues to be detecting EMT *in vivo*. Ideally, to prove the occurrence of EMT, one would need to view and track epithelial cells as they transition into fibroblasts and migrate to the interstitium in real time. Unfortunately, this is not feasible with current technology. Instead, one is left with compelling snapshots of the transitional process through histochemical analysis of fibrogenic tissue [23,31]. Additionally, several studies have demonstrated evidence of a partial EMT phenotype, identifying cells expressing both epithelial and mesenchymal markers in a diabetic setting [37,54,73]. This intermediate phenotype, *i.e.* cells that continue to express epithelial markers as new mesenchymal markers arise, is probably relevant since the acquisition of mesenchymal markers and the expression of ECM proteins occurring early in EMT may be transient and potentially reversible, at least in the early stage [7,39]. Thus, EMT is certainly a highly dynamic process that can lead to the formation of a fibroblast, but the transition does not necessarily have to carry through to a full fibroblast phenotype but may stop at various stages [23]. However, the two fundamental questions remain: whether type II EMT really occurs *in vivo* and, if yes, whether EMT does contribute significantly to interstitial fibrosis – in other words, is EMT essential for renal fibrogenesis in chronic kidney diseases in general, and in DN in particular [7]? As discussed in this review, the numerous EMT studies focus primarily on the initial EMT stages in mice as well as in human renal biopsies and cannot provide direct proof of the existence of EMT *in vivo*. However, taken together with studies that elucidated individual steps leading to invasion in the interstitium and to activated myofibroblasts, the evidence in favor of EMT should not be dismissed. On the other hand, the data from Humphreys’, Koesters’, and Faulkner’s cell lineage tracking studies, which fail to detect any evidence of EMT, appear highly conclusive. In particular the study by Koesters *et al.* that failed to detect any contribution of EMT to interstitial fibrosis in a strictly TGF-β-driven model, despite the fact that TGF-β1 has been considered to be a master regulator of EMT [74] appears to be conclusive. Furthermore, most studies use α-SMA expression in tubular epithelial cells and FSP1 expression in myofibroblasts as a representative marker for EMT, but some authors have argued that neither α-SMA nor FSP1 is a reliable marker because they lack cell specificity and constancy of expression [7,23]. Especially for FSP1—there is evidence that it may also be expressed in the leukocytes of injured kidneys. Therefore, it is justified to ask whether it can be considered a fibroblast-specific protein [23,69]. Intriguingly, FSP1 is also expressed by podocytes, raising the possibility that EMT proceeds in podocytes [69]. Given this, the fact that Humphreys *et al.* did not find any tubular cells that expressed α-SMA in UUO is remarkable, given that several studies of EMT in DN, as mentioned above, reported *de novo* α-SMA synthesis in rodents as well as humans. These discrepancies may be due to differences in the experimental design, underlining the complexity of the EMT process. It is the general consensus that EMT is an extremely dynamic process that seems to occur in a wavelike fashion, implying that correct timing is an important factor in the detection of EMT [7]. For example, Liu *et al.* demonstrated that the kidneys of their STZ-induced rats exhibited EMT, as illustrated by an early reduction of E-cadherin expression and a burst in fibronectin expression 2 weeks post-insult, but a markedly delayed upregulation of α-SMA 12 weeks after diabetes onset [29]. Interestingly, the late and/or rare expression of α-SMA has been also reported during human EMT [9]. The majority of EMT studies rely heavily on positive identification of transition-in-action cells, cells at a transitional stage coexpressing both epithelial and mesenchymal markers simultaneously, but the duration and/or fate of these presumptive intermediate transitional cells is unknown. Indeed, it has been argued that the transitional phase of EMT may be very short, rendering the timing of tissue analysis yet more critical for interpretation [7,23]. However, one can question whether the murine UUO model is a good model to recapitulate human fibrosis, which develops over a much longer time scale—it may take months for such changes to occur in mice, or years in humans [11]. For example, while the remodeling activity in the tubulointerstitial area of DN patients as mediated by myofibroblasts is most obvious in DN of class III and IV, which are late stages of human DN [24], Koesters *et al.* analyzed the fibrotic kidneys of their mice after only 4 days [70]. UUO, which recapitulates various key features of fibrotic responses including tubular damage, is indeed the most intensively used model, but it should be noted that UUO was also the model employed in the studies that failed to detect EMT. It remains to be determined whether the sequence of events in the UUO model can be applied to other types of kidney fibrosis or even reflect the time-course of human UUO [33,66]. Indeed, a recent study documents that the contribution of EMT to renal fibrosis differs among kidney disease models [75,76]. Inoue *et al.* speculate that differences in experimental conditions (including the disease model), background of the mouse strain, and type of genetic modification used may account for the discrepancies observed in the reports of EMT involvement in the setting of renal fibrosis [75,76]. To investigate this possibility, they generated kidney disease models in several mouse strains genetically modified to express enhanced green fluorescence protein (EGFP) in cortical tubular epithelial cells under the control of the γ-glutamyl transpeptidase promoter. Based on their findings, these investigators concluded that, at least in the mouse kidney, EMT promotes the development of interstitial fibrosis in both a disease-specific and strain-dependent manner. For example, using the UUO model, the same model employed by Humphreys *et al.*, Inoue and coworkers detected EGFP-positive interstitial cells. Furthermore, in the UUO model, EMT-related transcription factors were found to be upregulated. Importantly, the authors applied the UUO model to a fibrosis-resistant mouse strain and detected no GFP-positive cells, despite the occurrence of interstitial alterations, which was consistent with the results of previous studies that reported EMT only at extremely low levels in the UUO setting [75,76]. As mentioned above, more conflicting results can be found in the literature, probably reflecting the different methods used to define and detect EMT in various disease models [77]. Owing to this heterogeneity of techniques, mouse strains and disease models used to detect EMT should ideally be standardized [9]. For this, Zeisberg *et al.* propose to investigate a collection of criteria for EMT, which include at least one changing protein in each of the four key events, to increase the likelihood that everyone is studying the same phenomenon [51]. It is furthermore probably safe to use multiple rather than single markers simultaneously to detect the coordinated changes associated with EMT in the epithelial structures. Since the observation of epithelial cells transitioning into fibroblasts and migrating to the interstitium in real time is not feasible, Zeisberg and coworkers suggested that a combination of epithelial cell markers (e.g. N-cadherin, E-cadherin, ZO-1), possible lineage tags, cytoskeletal proteins (e.g. α-SMA, vimentin), and transcription factor signals (e.g. Snail, Slug, Twist, FOXC2), and perhaps the changing levels of discrete microRNAs might best report the presence of EMT *in vivo* [9,51]. A variety of biomarkers have been published to demonstrate EMT, some of which are acquired and some of which are attenuated during transition (a number of commonly employed markers are listed in the reviews of Zeisberg and Quaggin [51,77]). Quaggin *et al.* also suggest that the greatest promise for identifying type 2 EMT lies in analyzing a collection of markers [77]. In a similar fashion to Zeisberg, Quaggin *et al.* divided the indicators of EMT into four categories: changes in epithelial contacts, cell-associated ECM components (e.g. collagens, fibronectin, HSP47, integrins), transcriptional regulators, and cytoskeleton. They also suggest that detection of at least one feature within each of the four categories should suffice. In a recent study from our laboratory, in which we have analyzed two or more indicators within each of the categories suggested above, we could provide strong evidence for an important role of EMT in the experimental model of STZ-induced DN [2]. As argued by Simonson, two lines of evidence suggest that EMT functionally contributes to fibrosis in DN: 1) The same extracellular cues that induce EMT also increase fibrosis; and 2) inhibitors of EMT attenuate or block fibrosis and progressive kidney disease in animal models of diabetes [13]. It should be noted that, *in vivo*, tubular epithelial cells are more likely to be exposed to a plethora of mediators, each individual factor being probably present at lower concentrations compared to strictly controlled *in vitro* experiments [39]. On the other hand, under diabetic conditions tubular epithelial cells receive signals from a multitude of potential EMT inducers, which is likely not the case in the UUO model. The second argument of Simonson is based on strong experimental evidence, for example the observation that administration of BMP7 or HGF blocks tubular EMT and reverses chronic renal injury [3,12,61]. Also, it has been recently shown that metformin, a drug widely used for treating type 2 diabetes that acts primarily by lowering blood glucose levels, prevents tubular injury via inhibition of TGF-β-induced EMT [62,78]. These data strongly indicate that EMT is essential for renal fibrogenesis in chronic kidney diseases in general, and in diabetic nephropathy in particular. Still, the degree to which the EMT process contributes to kidney fibrosis remains unclear and is likely to be context-dependent. That is, dependent on the stage of fibrosis and the experimental model being studied [39]. Notwithstanding the fact that existing literature has not yet clearly defined the relative contribution of the different potential cellular sources of activated myofibroblasts, there is a general consensus that fibroblasts within fibrotic tubulointerstitium tissue have the ability to proliferate irrespective of their origin [23,75]. Considered together with the evidence that tubular epithelial cells, endothelial cells, and podocytes may all undergo phenotypic transition to promote the glomerular sclerosis and interstitial fibrosis in DN, this represents strong incidental evidence for a significant contribution of EMT and/or EndoMT to the pathophysiology of DN (notwithstanding that the contribution of EMT to myofibroblasts is less than 5% in DN, as reported by LeBleu *et al.* in the UUO model [32]). To somewhat defuse the debate, this review will close with a short summary of the perception of Galichon and Hertig, who recently stated that EMT could be a misunderstood and/or overrated concept [9]. Based on the limited power of currently available experimental techniques, like immunohistochemical approaches or gene expression analysis from total renal cortex, and since the *in vivo* studies do not allow for the investigation of all criteria defining EMT (especially the ability to migrate outside the basal membrane), the authors suggest the use of weaker terms such as ‘EMT-like changes’ or ‘partial EMT’ [9]. In their opinion, the term ‘EMT’ reflects more a global process in the renal tissue than local, molecular events occurring in epithelial cells [9]. Furthermore, they defined the term ‘mesenchyme’ as a synonym for ‘primitive connective tissue’, that is, any tissue that 1) is neither epithelial nor muscular nor nervous; and 2) the function of which is to provide an ECM used as a support for these other cell types to function properly [9]. Therefore, according to these authors, EMT is defined by phenotypic and functional properties that are reminiscent of mesenchymal cells rather than a full-way epithelial-to-fibroblast transition [9].

## 8. Conclusion

The reason for investigating the contributing role of EMT in tubulointerstitial fibrosis, which is the key pathology underlying DN, is the hypothesis that preventing interstitial fibrosis will slow the progressive decline in renal function. This review probed whether epithelial-to-mesenchymal transition in diabetic nephropathy is fact or fiction, a question largely overlooked in the past. There is much supportive evidence in the literature suggesting that EMT occurs not only *in vitro*, which is widely accepted, but also *in vivo*. Although solid studies argued against the role of EMT as a source of myofibroblast recruitment in fibrosis *in vivo*, we and others strongly suggest the existence of a direct relation between the number of myofibroblasts, TBM thickening and duplication, and interstitial fibrosis. Moreover, we do believe that EMT plays an important role in the development of DN. In addition, the sum of the data from the individual publications discussed herein convinces us that EMT and EndoMT do exist, at least to some extent, and that their role in the development of fibrosis in DN should not be underestimated. The exact percentage of renal epithelial cells or renal endothelia that become myofibroblasts in DN is entirely unknown. To get more insight, epithelial- and endothelial-tracing mice should be challenged in diabetic models such as STZ or genetic manipulation. Finally, the best fiction is based on good facts. Thus, there is no real contradiction.
